# Computational spectrometers enabled by nanophotonics and deep learning

**DOI:** 10.1515/nanoph-2021-0636

**Published:** 2022-01-24

**Authors:** Li Gao, Yurui Qu, Lianhui Wang, Zongfu Yu

**Affiliations:** State Key Laboratory for Organic Electronics and Information Displays, Institute of Advanced Materials, School of Materials Science and Engineering, Nanjing University of Posts and Telecommunications, Nanjing 210023, China; School of Electrical and Computer Engineering, University of Wisconsin-Madison, Madison, WI 53706, USA

**Keywords:** compressive sensing, deep learning, nanophotonics, spectral imaging, spectral sensing, spectroscopy

## Abstract

A new type of spectrometer that heavily relies on computational technique to recover spectral information is introduced. They are different from conventional optical spectrometers in many important aspects. Traditional spectrometers offer high spectral resolution and wide spectral range, but they are so bulky and expensive as to be difficult to deploy broadly in the field. Emerging applications in machine sensing and imaging require low-cost miniaturized spectrometers that are specifically designed for certain applications. Computational spectrometers are well suited for these applications. They are generally low in cost and offer single-shot operation, with adequate spectral and spatial resolution. The new type of spectrometer combines recent progress in nanophotonics, advanced signal processing and machine learning. Here we review the recent progress in computational spectrometers, identify key challenges, and note new directions likely to develop in the near future.

## 1 Introduction and overview

Optical spectrometers serve as one of the most important instruments in fields such as astronomy, biology, and chemical analysis [1, 2]. Conventional optical spectrometers typically consist of bulky dispersive elements such as gratings and prisms or interferometric elements. In conventional spectrometers like monochromators or Fourier-transform spectrometers, light only passes through the instrument once or twice, which means the optical path needs to be long enough to separate different wavelengths and achieve high spectral resolution. The long optical path, which is typically on the order of tens of centimeters, limits the size of the instrument. Recent demand for wearable electronics and portable instruments has stimulated enormous interest in smaller, cheaper spectrometers that can also provide real-time, accurate readouts [3], [4], [5], [6], [7], [8], [9], [10].

In contrast to conventional geometric optics, nanophotonic structures can increase the length of the optical path to millions of times larger than their physical size [11, 12]. Different materials and geometric designs of nanophotonic structures can induce completely different transmission, reflection, scattering, and absorption characteristics at every wavelength. Such strong light–matter interaction is referred to as the *response function* of nanophotonic structures. The response function of Fabry–Perot resonator is ideally a narrow-band delta function, while many periodic nanophotonic structures are broadband, with randomly distributed spectral features. The term *filter response functions* is commonly used since the transmission mode of operation is adopted more often. The micrometer-sized nanophotonic filters can be integrated directly onto pixels of photodetectors so that the spectral information can be encoded by precalibrated response functions and recorded in a snapshot by detector arrays. With the aid of appropriate computational algorithms based on compressive sensing (CS) theory, the spectral information can be accurately decoded and reconstructed [13–25].

Such emerging and novel spectroscopic technique is called *computational spectroscopy*. As no moving elements or bulky components are involved, such spectrometers can be extremely compact in size – with a footprint as small as sub-millimeters – and robust enough for portable or wearable application. Furthermore, the data acquisition in a snapshot and the data processing assisted by computation allows for simple, automated operations. Exciting innovations in both hardware and software solutions are merged in such systems, which are fundamentally different from conventional spectroscopy.

**Figure 1:**
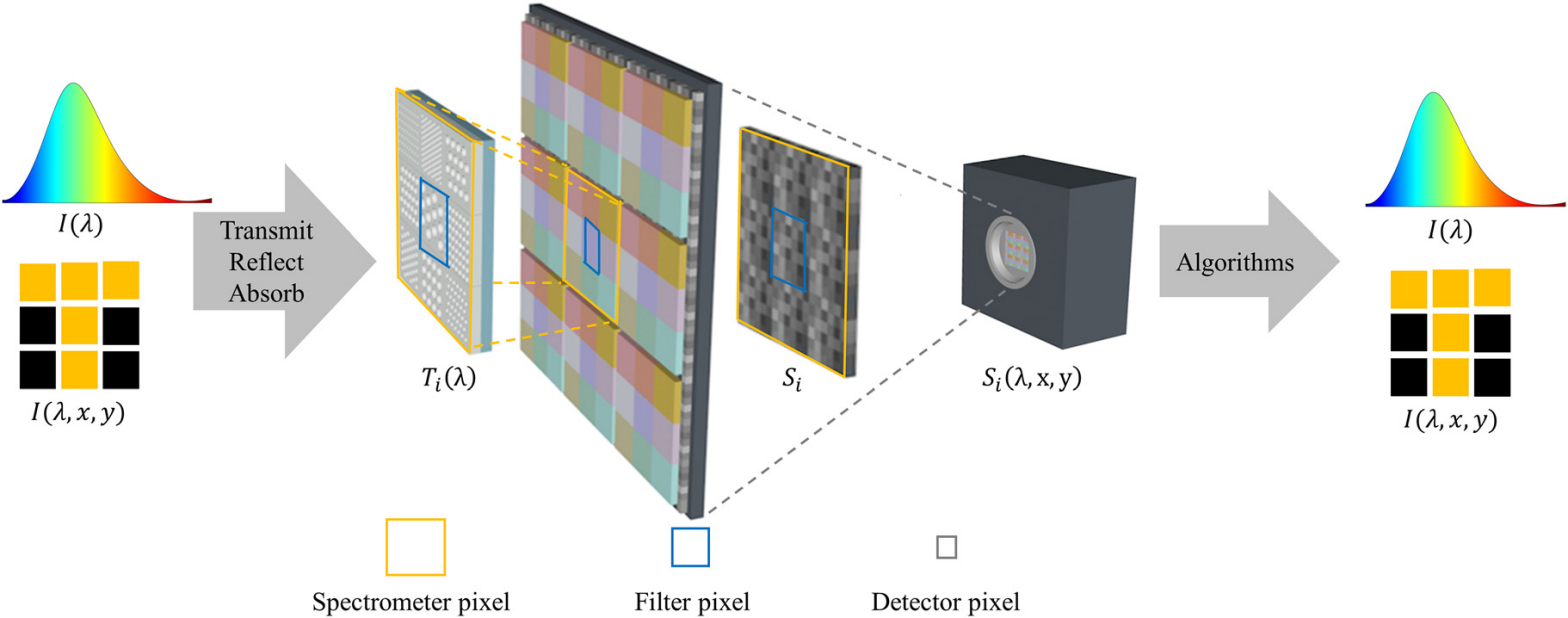
The major components and common operation mechanisms of computational spectrometers. Transmitted, reflected, or absorbed spectra are filtered by multiple nanophotonic structures, each with distinct response functions. The wavelength-dependent light signal is collected by sensor pixels and used for computational spectrum reconstruction.

The typical workflow of computational spectrometer consists of three steps: calibration, measurement, and the spectrum reconstruction process. The calibration process measures the response function of the optical filter array using a monochromator or a tunable laser. The measurement process is usually to capture a single-shot image of the unknown sample. The third step is to reconstruct the unknown spectrum based on the filter matrix and the single-shot image data from the first two steps, usually through CS theory and reconstruction algorithms based on minimizing regularized squares errors with non-negativity constraints [26–29]. The typical operation scheme for spectral sensing and imaging are sketched in Figure 1. A single spectrometer is often used for a single-point measurement without much spatial resolution. Therefore, for spectral sensing, a single spectrometer can perform a single spectrum I(λ) reconstruction.

In contrast, hyperspectral imaging technology allows both spectral and spatial information to be obtained simultaneously. In hyperspectral imaging, a 3D data cube is generated from a 2D spatial image plus a third dimension of spectral information, such as an RGB color image. Hyperspectral data cubes can contain absorption, reflectance, or fluorescence spectrum data for each image pixel. Therefore, for spectral imaging, a data cube I(λ,x,y) with both spectral and spatial information can be reconstructed.

Although the hardware set-up and data acquisition operations are straightforward and simple, the decoding process for interpreting the multiplexed spectral data is challenging. In the encoding process (the above-mentioned measurement process), the complex interaction between incident spectrum and nanophotonic filters forms the detected multiplexed signal data. For decoding, we must solve inverse problems using computational algorithms to solve a set of linear equations, provided that the parameters are carefully chosen and sufficient iterative computation time is available [30–35]. Conventional reconstruction methods use regularizations based on smoothness and sparsity to improve the reconstruction quality, but these methods often result in severe distortions in the reconstructed spectrum due to the ill-posedness of the problems under very noisy environments. As this research area evolves, the solution to the most challenging spectrum reconstruction problems is ideally found in integration with data-driven deep learning (DL) techniques [36–39]. With this approach, the specific filter response functions do not need to be precalibrated. Instead, a large, labeled dataset is fed in to the neural network for training and mapping the relationship between the spectrum and detected signal. Such a one-time investment in training procedure would enable numerous and instantaneous spectrum reconstructions at the subsequent application stage. The reconstruction accuracy and efficiency are no longer experience-dependent and can also be greatly enhanced with better noise tolerance. Therefore, machine learning tools can potentially dissolve the limitation of computational techniques and deal with big data problem, which can enable truly intelligent spectroscopic sensing and imaging systems with unprecedented performance.

In this review, we introduce computational techniques in spectroscopy where nanophotonics are involved as the hardware component in the encoding process and relevant computational algorithms as the software component used for decoding process. Later, we focus more on the fusion of DL techniques for spectrum reconstruction tasks. Please note that DL has been used intensively in nanophotonic inverse design problems; i.e., with desired resonant spectral features in mind, deep neural networks (DNN) can output the best set of nanophotonic design to produce the desired spectrum. This topic has previously been discussed in multiple comprehensive reviews [40–44] and is significantly different from our scope. In our current topic, we care about the application of computational and DL techniques in spectroscopic reconstructive processes, rather than using DL to design photonic components. With the implementation of DL tools, the spectral and spatial data can be well reconstructed for next-generation sensing and imaging technologies.

## 2 The encoding and decoding process in computational spectrometers

### 2.1 Nanophotonic filters as spectral encoders

Powered by the manufacturing ability to precisely sculpt the metallic and dielectric nanostructure geometry, light can easily be controlled to great distance with tailored light–matter interactions below the optical wavelength. It is fascinating that an ultra-thin, flat nanophotonic device composed of arrays of metallic or dielectric resonators can alter light propagation so effectively [45]. The field of nanophotonics has attracted significant attention from those working with light manipulation and photonic information technology. Examples include photonic crystal slabs fabricated with high-refractive index materials [13, 46–48]; plasmonics that confine light into an extremely small scale, resulting in huge near-field enhancement [49]; and metasurfaces that induce anomalous refraction and reflection by engineering the spatial and spectral response of the impinging wavefront [50, 51]. Therefore, careful selection of nanophotonic geometry, materials, and unit cell arrangements can excite the desired resonance with incident light, resulting in impactful applications such as high-quality structural colors [52], ultraflat metalens [53, 54], high-density data storage units [55], etc. Moreover, nanophotonics have been used as color filters and polarizers in image sensors [56].

**Figure 2:**
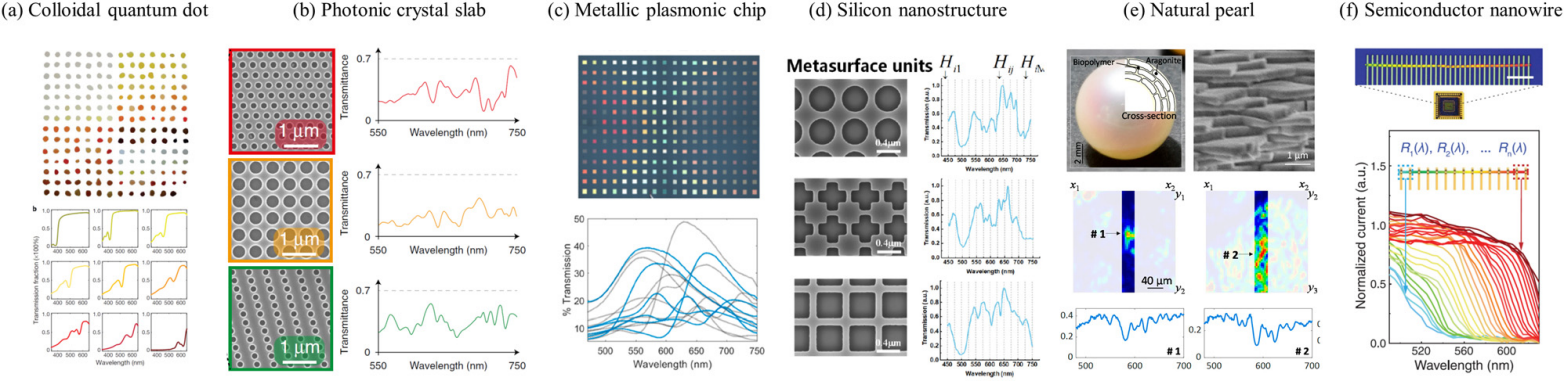
The response functions generated by different nanophotonic structures. (a) 195 colloidal quantum dot materials in the form of filters. Each dot is a CQD filter made of one type of CQD material embedded in a polyvinyl butyral thin film, with the representative transmission spectra for some of the CQD filters shown below. (b) Three scanning electron microscopy (SEM) images of selected PC-slab structures marked by red, orange, and green frames on the left and corresponding measured transmission spectra *T*(*λ*) of the three structures on the right. (c) Brightfield microscope image of the plasmonic encoder chip showing example transmission spectra *T*(*λ*) below. (d) Illustration of three different silicon metasurface units and their response functions, used for reconstructive spectral imaging. (e) Cross-sectional and SEM illustration of the brick-and-mortar nanostructure of pearls. Crystalline nacre is composed of aragonite platelets bound together with organic macromolecules (biopolymer). (a) Hyperspectral line-scanning along the vertical direction (middle) is acquired to measure the corresponding transmission spectra of selected spots (bottom). (f) Fluorescent micrograph (top) of a typical nanowire spectrometer incorporated into a packaged chip. Normalized spectral responses of each constituent unit in a typical spectrometer (bottom) with cutoff wavelengths varying continuously along the nanowire. (a) is reprinted with permission from Ref. [19], copyright 2015 Nature Publishing Group; (b) is reprinted with permission from Ref. [22], copyright 2019 Nature Publishing Group; (c) is reprinted with permission from Ref. [38], copyright 2021 American Chemical Society; (d) is reprinted with permission from Ref. [27], copyright arXiv.org; (e) is reprinted with permission from Ref. [64], copyright 2021 American Chemical Society; (f) is reprinted with permission from Ref. [65], copyright 2019 AAAS.

The origin of intense light–matter interaction in periodic nanophotonics is due to the light bouncing back and forth with very enhanced optical paths at interfaces that modify the collective amplitude, phase and polarization properties of light propagation. The diverse design of nanophotonic filters, such as cavity and resonator filters, can theoretically result in a *Q* factor of ∼10^9^ [12] and an experimental *Q* factor of more than 2 × 10^6^ [11] at specific resonant frequencies. However, the range of application wavelengths is limited to narrow bands of resonance. Such highly resonant nanophotonic designs are desired in narrow-band filters, spectrometers and those spectral-sensing applications which require narrow, sharp resonance peaks with high *Q* factors; thus precise spectrum shifts caused by small traces of analytes are easy to detect [29, 57, 58]. In earlier experiments, high-*Q* resonance Fabry–Perot and etalon-array types of structures have also been used as band-pass spectral filters for computational spectrometers [20, 31]. In general, these resonant filters have simple Lorentz line shapes and lack the spectral diversity to provide high-spectral resolution. In order to pursue high-spectral resolution, a number of filters with sharp passing bands are usually required, which is practically challenging due to the design and fabrication complexity [32, 47, 59–62]. To resolve this dilemma, researchers turn to arrays of flat nanophotonic filters that can induce broadband, wavelength-dependent and diverse resonant features to encode the unknown spectrum. Such nanophotonic filters are advantageous when compared with conventional narrow-pass band filters, because they can expand the operational range with high-spectral resolution. The production of these filters is also compatible with nearly all photonic materials and structures [63]; some typical examples are discussed in Figure 2.

As illustrated in Figure 2a, a diverse range of random spectral features can be created by absorption in colloidal quantum dots (CQD) filters [19]. The absorption spectra can be tuned continuously and finely over wavelengths ranging from deep ultraviolet to mid-infrared simply by changing the CQD’s size, shape and composition. In contrast to quantum dots, where the fabrication could be complicated, photonic crystal (PC) slabs can be defined via single-exposure photolithography and only require standard CMOS materials [22, 23]. In PC slabs, the light can bounce in a lateral direction parallel to the slab into the interfaces of air holes, forming guided resonances with high-quality factors. The light can also bounce along interfaces in a normal vertical direction to the slab and form Fabry–Perot resonances with low-quality factors. The combination of multiple resonances can enhance the diversity of spectral features through Fano interference [66, 67]. Three representative examples of different PC slabs and their corresponding spectra are shown in Figure 2b, where multiple random resonance features are present. As the spectral response functions are entirely extrinsic and enabled by structures instead of materials’ properties, the concept can be applied to other wavelength ranges by scaling the dimension of PC properly. Other than perfect periodic photonic structures, filters can also be made based on the semi-regular Voronoi structure, which forms multilayered photonic crystals with random wave-like structures in each of the Voronoi cells. The transmission characteristics of the multilayered photonic crystal can be controlled simply by changing the cell’s microstructure, and there is no need to change the manufacturing process and materials. Furthermore, such Voronoi cells can be randomly distributed, so the filter can be positioned at arbitrary positions and angles, followed by filter-response characterization after mounting [68].

Other than photonic crystals, metallic plasmonic structures are another important type of broadband filters. An array of plasmonic metasurface filters was fabricated by e-beam lithography and used for short- and long-wave infrared spectra reconstruction [21]. One can also utilize a flat spectral encoding chip with an array of nanostructured plasmonic tiles to filter the incident light, where each tile has a specific transmission spectrum, as shown in Figure 2c. The broad maxima and resonance features result from the plasmonic modes supported by the metal nanostructures. Such filter chip can be fabricated through a scalable and low-cost imprint lithography process with metal deposition [38].

During the past few years, dielectric metasurfaces have been examined intensively. Here, meta-atoms can effectively alter the light phase and polarization with much less energy loss than metallic structures. Ultrathin and high-performance metasurfaces are compatible with CMOS fabrication [69] and can replace bulky geometric optics [51]. The thin, compact form of dielectric metasurfaces is also ideally suited for transmission filter responses. Changing the geometry of dielectric metasurfaces can result in sensitive optical resonance features. For example, a planar, array-like geometry of silicon nanostructure shows unique scattering spectrum depending on whether a silicon block is present. Such scattered spectrum response can be used for optical data information storage [55]. A C4 symmetric metasurface design that is formed on silicon-on-insulator chips and later transferred onto CMOS image-sensor chips is proposed for polarization-independent performance with expanded spectrometer applications. A total of 400 different filters are fabricated; three representative examples are shown in Figure 2d.

On the contrary to manmade devices, where randomness and diversity are introduced on purpose, a natural or cultured pearl has a large number of alternating layers of crystalline aragonite and organic macromolecules with an irregular brick-and-mortar structure, as seen in Figure 2e. The low-dimensional irregular nanostructures found in pearls can be ideal for constructing highly uncorrelated spectral resonance via strong light localization to demonstrate spectral compressive sampling. Different areas of the pearl shows distinct intensity speckle maps and transmission spectrum [64]. Pearls thus provide a clue for simple, scalable, man-made filter components by carefully introducing the degree of disorder. With the recent advancement in materials growth and optoelectronic device design, it has also been demonstrated that a passive nanophotonic filter component and an active photodetector pixel can be integrated into one single nanowire device (Figure 2f), which means the spectral multiplexing and detection can be completed simultaneously [65]. In this pioneering work, an array of single nanowires can be engineered with a compositionally graded semiconducting CdS_*x*_Se_1−*x*_ nanowire, which corresponds to a continuous gradient of bandgaps spanning from 1.74 to 2.42 eV along their length, and results in different spectrum response functions. The photodetector units are defined as units between two neighboring electrodes, which can measure the photocurrents at different wavelengths. Such nanowire schemes serve both as spectral filter and photodetector, which allows further miniaturization of compact spectrometers. Similarly, a black phosphorous single spectrometer has leveraged the wavelength and bias-dependent responsivity matrix learned from the spectra of a tunable blackbody source. Unknown monochromatic and broadband spectra can be reconstructed from their corresponding photoresponse vectors in the mid-infrared range [70].

**Figure 3:**
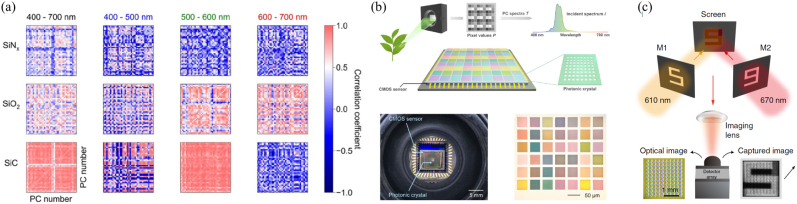
The correlation map between different nanophotonic filters and data acquisition set-up for spectral reconstruction and spectral imaging based on photonic crystal slabs. (a) Mapping of correlation coefficients between each pair of the 42 simulated transmission spectra in different wavelength ranges. Blue indicates weak or negative correlation; red indicates strong positive correlation. (b) Sensor operating principle and schematics (top). The spectral sensor consists of an array of PC slabs atop a CMOS image sensor. The transmission spectra of the PC slabs differ significantly from each other and form a sampling basis, T. For an incident light, the pixel values of the sensor and the sampling basis T are used to infer the unknown spectrum of the light. Device photograph (bottom) of an array of PC slabs fabricated on a SiN_x_/quartz substrate and placed on a CMOS image sensor. (c) Hyperspectral imaging using the PC spectrometer. The imaging target is a mix of the numbers “5” and “9”, with the “5” comprising light incident on the screen at 610 ± 5 nm and the “9” comprising light at 670 ± 0.7 nm. The spectral information of this target is obtained via a single snapshot. The optical and captured images are shown on the left and right of the device, respectively. (a) and (b) are reprinted with permission from Ref. [23], copyright 2019 Optical Society of America; (c) is reprinted with permission from Ref. [22], copyright 2019 Nature Publishing Group.

Reconstructing accurate spectrum by CS theory relies on the randomness of the measuring bases. Therefore, each filter response function should have diverse spectral features with both broad and narrow line shapes, as discussed. In addition, different response functions in the same spectrometer should have minimal correlation. To quantify such effect, a correlation coefficient r value measures the similarity between each pair of the nanostructure transmission spectra at different wavelengths. A value smaller than 0.5 indicates a moderate or weak correlation between different filter structures, and thus good designs. It is calculated by $r_{ij}=E\left\{\left[t_{i}–E\left(t_{i}\right)\right]\cdot \left[t_{j}–E\left(t_{j}\right)\right]\right\}/\left(\delta_{i}\delta_{j}\right)$ for each pair of rows in the transmission matrix in different wavelength ranges, where $t_{i}$ is the transmission of the i th filter, E indicates the average, and δ indicates the standard deviation [23]. Both material property and nanophotonic geometry determine the response functions and their correlation. For example, different PC slab filter designs have been investigated for their response function correlation for the entire 400–700 nm spectral range, as shown in Figure 3a. The mean values of the correlation coefficient are 0.84, 0.49, and 0.35 for PC slabs fabricated using SiC, SiO_2_, and SiN_*x*_. These results indicate the transmission spectra of SiC PC slabs are highly similar over a broad wavelength band, while SiN_*x*_ PC slabs show minimum similarity and most randomness in their spectra features. The undesirable high correlation in SiC is due to the strong absorption in the shorter wavelength range, while the correlation in the SiO_2_ PC slabs increases significantly in the longer wavelength range because due to a smaller refractive index, they can support fewer number of optical modes in the long wavelength range. It is found that the SiN_*x*_ PC slabs maintain low mutual correlation values in all spectral ranges and is most promising for spectral reconstruction. In the pearl spectrometer case [64], spectral similarity with high correlation coefficients (>0.5) is also avoided at adjacent transmission spots that couple to each other. The pairwise comparison of different transmission spectra are generally uncorrelated, with an average correlation coefficient of 0.161, which proves such naturally occurring irregular and random multilayers are desirable and may inspire similar biomimetic filter structures.

### 2.2 Spectral data acquisition

In a computational spectroscopic system, the spectral data acquisition and processing is mainly based on the concept of compressive sensing. Compressive sensing is digital-signal processing that offers the potential of high-resolution readout of sparse physical signals from fewer measurements than the Nyquist–Shannon sampling theorem requires [71–74]. The unknown spectrum is measured by projecting it to a set of random bases. Typically, the number of the measurements is smaller than the dimension of the unknown spectrum. The spectrum can be recovered by finding solutions to an underdetermined linear system. It is particularly powerful for recovering sparse signals using a fewer number of measurements. L_1_ norm regularization is used to force the unknown signal to be sparse [75]. In practice, the unknown spectrum signal is projected to random bases through the broadband nanophotonic filters. Random response functions are preferred to improve the spectral resolution, as the correlation between the functions is minimized. In general, whether a system is over- or under-sampling is determined by whether the number of detected signals is higher or lower than the number of data points in the reconstructed spectrum, respectively. As seen in many previous studies [22, 23, 27, 61], by just sampling tens of filtered detector responses, a band of a few hundred nanometers can be reconstructed with approximately 1 nm resolution: a compression ratio better than 10:1. This reduced sampling is obviously superior to a monochromator-based conventional spectrometer, where high spectral resolution must rely on high-precision gratings or tunable narrow-filter bands that sample hundreds of times.

In a typical setup of CS-based computational spectrometer (shown in Figure 1), a set of nanophotonic filters are coupled or integrated onto detector arrays made of charge-coupled devices (CCD) or CMOS technology. After a spectrum shines on the nanophotonic filters, transmitted, absorbed or reflected light intensity is recorded on the detector array underneath each filter. All these filters define the size of a single spectrometer, which may be considered as “spectrometer pixel” or “super pixel.” On the spectrometer pixel, there are multiple filters containing different nanostructures with distinct transmission, absorption or reflection response functions, and the size of a single filter is defined as “filter pixel.” Under each filter pixel, there are a few photodetector pixels that detect the signal; each one of them is called “detector pixel” or “subpixel.” In a single snapshot, multiple spectral measurements can be acquired without scanning or switching filters, and all spectral intensity data are recoded and used to reconstruct the incident spectrum.

(1) $$S_{i}=\overset{\lambda_{n}}{\underset{\lambda_{1}}{\int }}I\left(\lambda \right){\text{T}}_{i}\left(\lambda \right)\eta \left(\lambda \right)\mathrm{d\lambda},i=1,2,\ldots ,\text{ M}.$$

The above equation describes the mathematical operation mechanism of the spectra encoding process. Here, I(λ) is the spectrum of incident light source or the unknown spectrum signal to be probed. η(λ) is the spectral responsivity of the photodetector, which can be taken into account and measured in experiments. The signal $S_{i}$ , is the measured signal by the detectors underneath the i th filter, among the total M filters. Since the detector pixel size is usually smaller than the filter size, the signals from all the photodetector pixels are summed for each filter and normalized by the pixel sensor signal without filter. The total M filters and all associated detectors form one spectrometer pixel to collect all necessary signal $S_{i}$ . Most importantly, before the reconstruction of the unknown spectrum, the spectral response functions of the nanophotonic filters need to be calibrated first. This is usually done by using a wavelength-tunable light source incident from the normal direction with a spectral resolution of 1 nm or less. Thus $T_{i}\left(\lambda \right)$ is the different transmission spectra or response function produced by the i th filters, where λ is the free-space wavelength with the sampling number of N .

To apply the CS algorithm, we need to digitize all continuous spectral functions and transform Eq. (1) to a set of linear equations.

(2) $$S_{i}\approx \overset{\lambda_{n}}{\underset{\lambda =\lambda_{1}}{\sum }}I_{\lambda }{\text{T}}_{i,\lambda }\eta_{\lambda }=I_{1\times n}T_{m\times n}$$

The transmission matrix $T_{m\times n}$ ( M different nanophotonic filters at N different wavelengths) is a collection of different response functions of optical filters including the factor of η(λ). The i th row of the filter matrix $T_{m\times n}$ is the transmission spectrum of the i th filter. The filter matrix needs to be calibrated first, and is then used in the spectrum reconstruction process in formula TI=S , where S is the measurement and I is the target spectrum signal. The measured signal $S_{i}$ is collected by the photodetector array. The unknown $I_{1\times n}$ can be solved numerically. It is always desirable to digitalize the unknown signal with a large N for fine resolution. Therefore, CS focuses on the case N > M, and the linear equations are underdetermined. The unknown I(λ) can be recovered by multiple computation algorithms (discussed in the next section).

As shown in Figure 1, if spatio-spectral information such as the “T” letter is to be probed, multiple spectrometer pixels such as 3×3 spectrometer array can be integrated on the photodetector surface so the signal captured in a snapshot can reconstruct the spectral information at multiple locations that forms a spatial image. Therefore, the snapshot hyperspectral imaging applications are realized if multiple spectrometers are placed on the detector surface to record spatio-spectral signal $S_{i}\left(\lambda ,x,y\right)$ . Hyperspectral imaging is more challenging due to the large data cube sampled and processed with both spectral and spatial variation. The size of each super pixel defines the spatial resolution of the hyperspectral imaging. A high spectral resolution requires more filters in a single super pixel, but increasing filter numbers means a large super pixel size and low spatial imaging resolution.

Let’s look at a typical example of such a spectrometer system [22]. In the PC slab work shown in Figure 3b and c, the signal light of interest is in the spectral range from 550 to 750 nm. The spectrum is digitalized with 1 nm resolution, represented by N=201 unknowns to be determined. Each photonic crystal slab forms a single-filter pixel size of 32×32 μm. M=36 different PC slab filters defining a super pixel size of 210×210 μm. A similar work completed by the same group [23] has also demonstrated that a super pixel can be defined by just 3×3 filters and successfully recovers 301 spectral bands from 400 to 700 nm. The demonstrated PC slab hyperspectral imaging uses 10×10 super pixels, each composed of 36 filters with ∼200 μm size on a side, which successfully identifies the spectral and spatial information of simple digits at different wavelengths. Although this spatial resolution is very coarse (∼200 μm), they could be improved theoretically to be better than 10 μm if the filter size is reduced to a few micrometers and only 9 filters are used. A more recent work reported that the metasurface-based spectrometer can reconstruct spectrum with 25 filter units with a size of 80 μm×80 μm , covering a 300 nm broad visible spectrum with an ultra-high center-wavelength accuracy of 0.004 nm and spectral resolution of 0.8 nm [27]. Most importantly, in this study, a one-shot ultraspectral imaging device fitting 6336 pixels of micro-spectrometers on a chip no larger than 0.5 cm^2^ has been demonstrated.

### 2.3 Computational algorithms as spectral decoder

The nanophotonic filters encode the input spectrum I(λ) using distinct filter functions while reconstruction algorithms are then tasked to recover the incident spectrum from the raw data sampled by detectors. As discussed in the previous section, the most common approach to algorithmic reconstruction is to use *a priori* information on the encoding operation (or the response function) $T_{i}\left(\lambda \right)$ and spectral sensitivity of the photodetectors η(λ) to define a transformation between the target spectrum I(λ) and raw measurements $S_{i}$ , that is, $S_{i}=T_{i}\left(\lambda \right)\eta \left(\lambda \right)I\left(\lambda \right)$ for each i th encoding operation. The reconstructed spectrum should minimize the sum of the squares of the differences between measured light intensities and numerically computed intensities, which are the products of the reconstructed incident spectrum and the filter response function, which are measured for each filter and integrated with respect to wavelength. By expressing this transformation operation over all the encoding elements, a least squares linear regression problem can be defined, and a solution for I(λ) can be obtained by minimizing ∥ S−TηI ∥^2^.

However, the inversion problems presented above are typically ill-posed with associated measurement error and noise. Standard least squares solutions are limited and insufficient to solve the underdetermined problem. Therefore, regularization techniques and constrained optimization methods are necessary to overcome the limitations and solve the inverse problem properly. Some more popular reconstruction algorithms are regularization-based algorithms, such as non-negative least squares [30], Tikhonov regularization (TR) [32], and sparse recovery based algorithm [15, 33–35]. There are several widely used regularization terms in CS spectrometers. One regularization term is L_1_ norm $∥I{∥}_{1}$ of the signal I . Minimizing L_1_ norm forces the signal I to be sparse and is well-suited to signals that can be efficiently represented in a relatively small number of measurements in spectrometers. It is also a convex optimization problem that can be reduced to a linear program known as basis pursuit [16, 20, 74]. The other is the second-order derivative of the spectrum signal $∥DI{∥}_{2}$ , where D is the second-order derivative of the spectrum signal I . Minimizing $∥DI{∥}_{2}$ forces the spectrum signal I to be smooth. The method of TR based on L_2_ norm minimization was also introduced to solve the spectrum reconstruction problem by careful regularization parameter selection. Different regularization parameters, such as the L-curve criterion and generalized cross validation criterion, have been examined [26]. This method can be used across different spectrometric devices of different filtering characteristics without any manual calibration of parameters. It was also reported that TR is able to account for experimental noise by suppressing divergences in the singular values below a critical noise parameter. CS based on L_1_ norm minimization produces relatively low computational reconstruction noise and also allows the recovery of a more complex spectrum than TR reconstruction due to its ability to suppress background computational noise.

**Figure 4:**
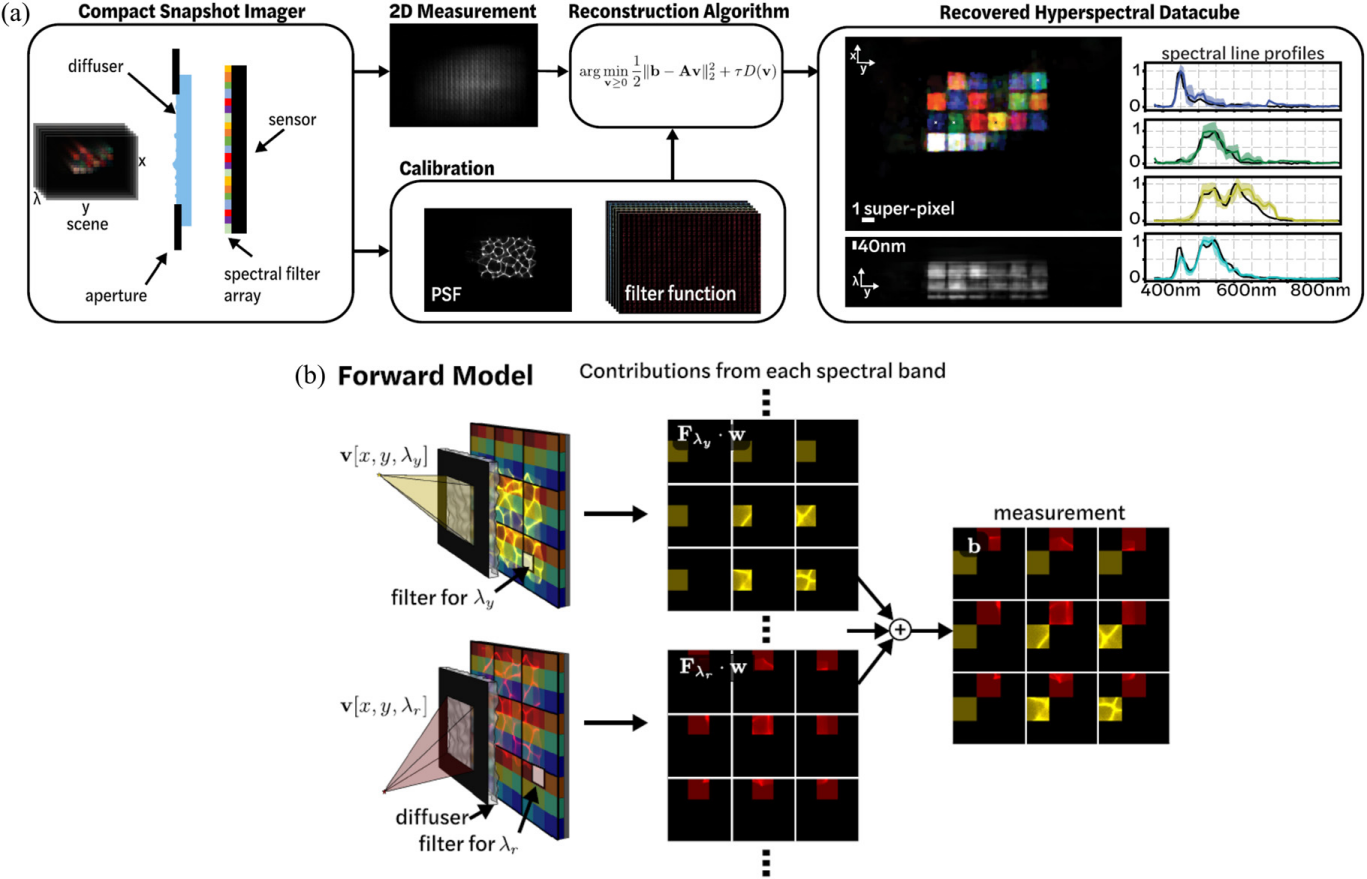
The working mechanism of combining a diffuser and spectral filter array to improve the spatial resolution in spectral imaging. (a) Overview of the Spectral DiffuserCam imaging pipeline, which reconstructs a hyperspectral data cube from a single-shot 2D measurement. (b) Image formation model for a scene with two point sources of different colors, each with narrow-band irradiance centered at y (yellow) and r (red). The final measurement is the sum of the contributions from each individual spectral filter band in the array. (a) and (b) are reprinted with permission from Ref. [28], copyright 2019 Optical Society of America.

The reconstruction of spectral snapshot compressive imaging can be more complex, as it involves large spatio-spectral data cubes. Generally used regularizers or priors for spectral snapshot compressive imaging include the sparsity [76], total variation [77], patch-based methods such as dictionary learning [78, 79] and Gaussian mixture models [80]. By utilizing the nonlocal similarity in the spectral data cube, group sparsity [81], and low-rank models [82] have also been developed to achieve state-of-the-art results in snapshot compressive imaging. Recently, a new encoding and decoding scheme has taken advantage of both spectral filters and lensless imaging to achieve spatio-spectral high-resolution snapshot hyperspectral imaging [28]. This system consists of a tiled spectral filter array placed directly onto the sensor and a diffuser placed a small distance away from the sensor, which is termed the DiffuserCam architecture [83] shown in Figure 4a. The system consists of a diffuser and spectral filter array bonded to an image sensor. A one-time calibration procedure measures the point spread function (PSF) and filter function. The diffuser spatially multiplexes the incoming light, and each spatial point maps to corresponding pixels on the camera. The spectral filter array then spectrally encodes the incoming light such that the exposure on each pixel is the sum of point-wise multiplications with the discrete filter function. The multiplexing effect of the diffuser allows recovery of scene information from a subset of sensor pixels. Figure 4b shows the image formation model for a scene with two point sources of different colors, each with narrow-band irradiance centered at y (yellow) and r (red). The final measurement is the sum of the contributions from each individual spectral filter band in the array. Due to the spatial multiplexing of the lensless architecture, all scenes project information to multiple spectral filters; that is why a high-resolution hyperspectral cube can be recovered from a single image after solving an inverse problem. To recover the hyperspectral data cube from the 2D measurement, the authors also use L_1_ minimization within the framework of CS to solve an underdetermined inverse problem with a prior to sparsity [84, 85]. The result is a 3D hyperspectral cube with 64 channels of spectral information for each of 448,320 spatial points, generated from a 2D sensor measurement that is 448,320 pixels. In the experiment, the authors built a prototype and demonstrated reconstructions of complex spatio-spectral scenes, achieving up to 0.19 super pixel spatial resolution across 64 spectral bands, which performs better compared to previous studies based on only spectral CS.

## 3 Spectrum reconstruction enabled by deep learning

### 3.1 Introduction of deep neural networks

The successful adoption of computational algorithms to reconstruct spectrum requires *a priori* calibration of filter response functions and careful selection of algorithm operations. Thus, the reconstruction process is generally iterative and time consuming. DL algorithms are part of artificial intelligence (AI), which can perform supervised learning tasks through data-driven approaches. With the invention of new training and regularization techniques, such as activation and loss functions, dropout and batch normalization, DL can now exploit large and complex datasets with unprecedented capability [86–88]. In general, DNN consists of a large series of interconnected processing nodes or neurons that connect the inputs to the outputs through a series of intermediate layers. The large number of internal parameters that can be optimized during the training offers high plasticity to learn complex and nonlinear relationships within the training data. Another distinct advantage of DNN is that a wide variety of architectures can be employed to model the specific data more efficiently and accurately [89–92].

The rise of DL tools has reformed multiple interdisciplinary research topics and enabled manifold applications which formerly seemed strictly impossible. A prominent example is a data-driven approach to solve various inverse problems, whereas conventional methods are computationally expensive and slow. The photonics community benefited from such rapid advances in DL techniques, especially in nanophotonic inverse designs [93–95]. Unlike conventional optimization that explores the design space iteratively and consumes lots of computing resources and time, DNN can produce fast and accurate forward predictions that can replace numerical simulations and realize direct inverse design of photonic devices. For example, by leveraging inverse design capability of DL techniques, sensor hardware can be fundamentally redesigned to obtain the optimal sensing data with respect to a user-defined cost function or design constraint. Thus a new generation of computational sensing systems with improved sensing capabilities are also emerging [96]. Well-trained DNN models can directly map the complex structure-response relationships in various scenarios so that the cost of one-time training can allow orders-of-magnitude efficiency improvement for problem solving. DL technique has previously proved promising in decoding complex data, as it enables rapid and accurate analysis of optical spectra and images [97]. Moreover, it has also become evident that DL architectures can be effective for computational image formation from as well as interpretation. The DL-based imaging approach has proven to be especially useful when the image measurement is noisy and the measurement operator is ill-posed or uncertain [98].

**Figure 5:**
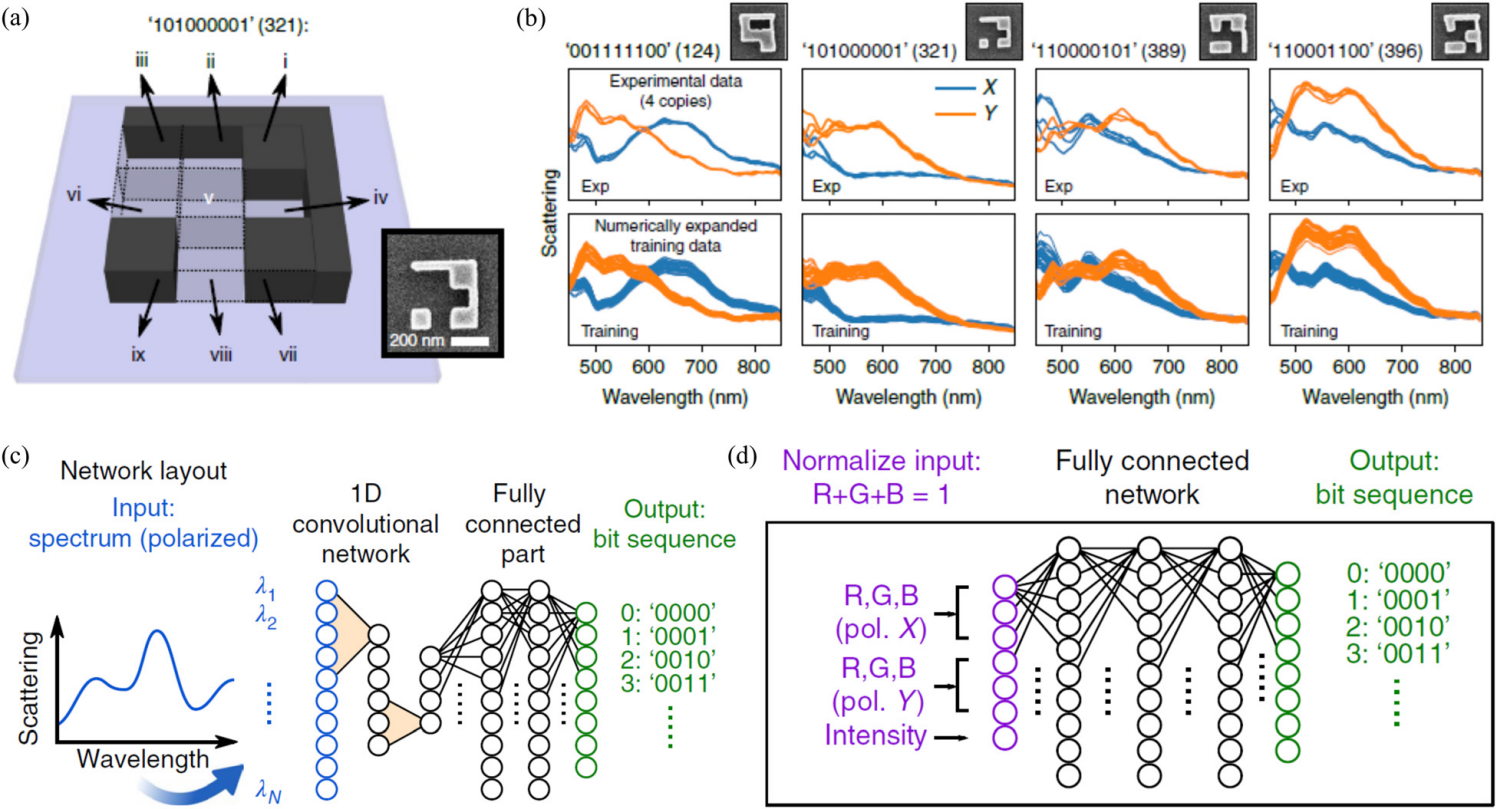
The application of DNN to decode nanophotonic spectral encoded optical information. (a) Geometry of a silicon nanostructure encoding 3 × 3 = 9 bits. Each silicon block occupies an area of 105 × 105 nm^2^. The L-shaped sidewall is 45 nm wide, and the height is 90 nm. An SEM image of a fabricated structure is shown in the inset. The example shown represents the decimal number ‘321.’ (b) Selected examples illustrating training data generation by a numerical expansion of the experimental spectra. SEM images show areas of 550 × 550 nm^2^. A large set of spectra is generated via random superposition of the experimental scattering spectra of these four copies to allow training and testing the performance of the binary information readout ANN. (c) Sketch of the 1D convolutional neural network used for the classification task, measured spectra and corresponding digital information are fed into the network. The error is back-propagated using a variant of the stochastic gradient descent (SGD) algorithm. (d) Scheme of the fully connected ANN used for the RGB classification task. (a–d) are reprinted with permission from Ref. [55], copyright 2019 Nature Publishing Group.

There is growing interests in applying DNN to computational spectrometers. Since DNNs can operate based on data-driven and “black box” learning processes, they do not require priori knowledge of the filter response functions to reconstruct the spectrum. Instead, they can learn the response functions from large spectra-signal datasets and directly map the relationship between spectrum and measured signal. Although the early work shown in Figure 5 was not targeted for spectrum reconstruction, it demonstrated that DNN can effectively learn spectrum data features and map the complex resonant spectrum to corresponding bit sequence readouts. In this work, a 1D convolutional neural network followed by a fully connected network is used. The spectra are fed into the network input layer, which consists of one or two parallel channels depending on whether one or both polarization cases are used. At the output layer, each neuron with the highest activation indicates the encoded bit sequence. Therefore, DL algorithms can read far-field spectrum encoded information accurately. Intuitively, by feeding sufficient training data to DNN with proper training, we can also directly map the relationship between incident spectrum and filtered snapshot signals without knowing the exact filter response functions. After a one-time training, the unknown spectra can be reconstructed instantly by inputting the measured photodetector signal. The performance of the DNN-based computational spectrometer is often evaluated by the mean square error (MSE) between the predicted spectra and the ground-truth. Since the spectral intensity value is in the range of 0–1, an MSE value on the order of 10^−4^ to 10^−5^ indicates a good reconstruction performance [35, 99–105].

### 3.2 DNN-assisted algorithm for spectrum reconstruction

DNN opens up new opportunities for designing accurate and robust algorithms for spectrum reconstruction and hyperspectral imaging. A natural and intuitive idea to utilize DL for spectrum reconstruction is to train a multilayer perceptron just like those in Figure 5, which takes pairs of raw measurements and the true spectrum as its input and output. Unlike the regularized reconstruction methods in which an explicit mathematical formulation of the underlying model is needed, DNN uses a black box to map the relationship between the raw measurements and the true spectrum. The application and training of CNN can predict spectra from the feature extracted by applying a direct inversion operator for CS computational spectrometers [104]. A residual CNN is proposed to deliver better reconstruction performance than CNN and sparse recovery [105]. These research findings demonstrate the potential of DL to facilitate spectrum reconstruction in ultracompact spectral imaging and sensing systems.

**Figure 6:**
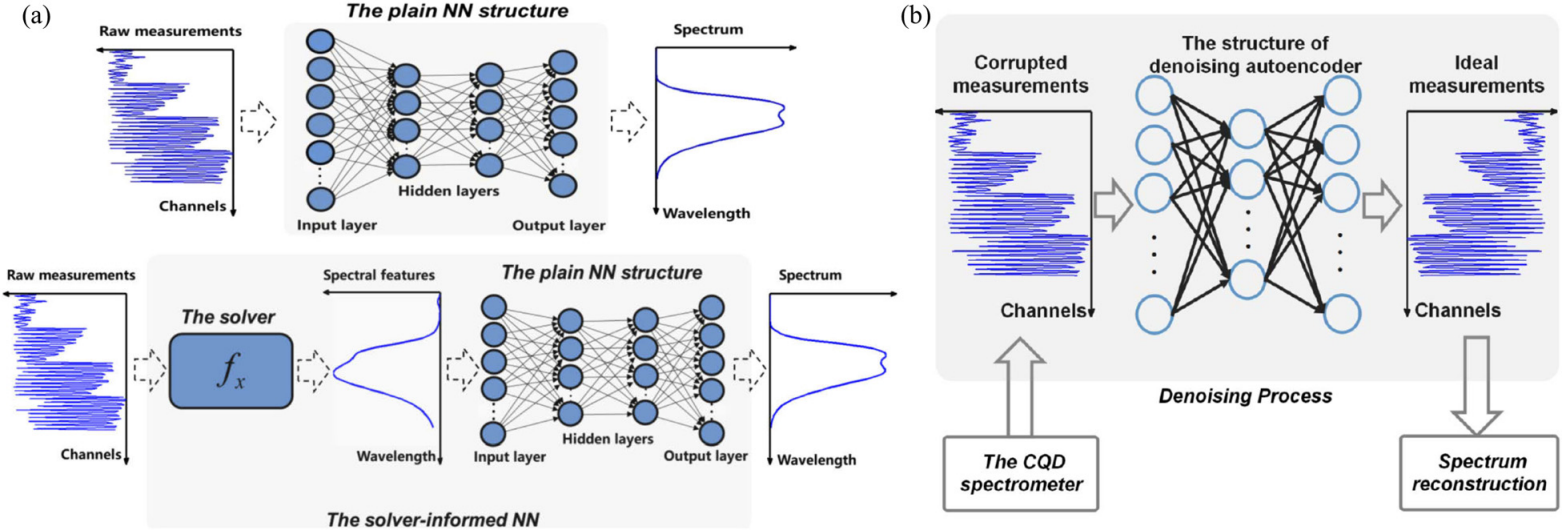
The proposed solver-informed NN and denoising autoencoder architecture used for spectrum reconstruction. (a) The architecture of the plain NN (top): taking pairs of raw measurements and the true spectrum as input and output, the flowchart of the solver-informed NN (bottom) includes applying a specific spectrum reconstruction solver to extract spectral features from the raw measurements and using the spectral features as an input of the NN to learn a map from the features to the true spectrum. (b) The denoising autoencoder architecture with one pair of input and output. The input and output layers match the corrupted and ideal measurements of the CQD spectrometer. (a) is reprinted with permission from Ref. [36], copyright 2021 Optical Society of America; (b) is reprinted with permission from Ref. [37], copyright 2021 IEEE.

Instead of using the direct inversion operator, Bao et al. [36] employed an existing spectrum reconstruction solver to extract spectral features from the raw measurements of CQD filters and then use the extracted features to train the DNN to learn a map from the features to the ground-truth spectrum (which is referred as solver informed NN). The architecture is compared with the plain NN based on raw measurements, as shown in Figure 6. The plain NN is a typical architecture composed of an input layer, several hidden layers and an output layer together with a nonlinear activation function. An appropriate loss function such as MSE is chosen to train a NN model. Such standard DNN training method is straightforward, and the specific training instructions can be found in relevant works [36, 93–95]. The plain NN outperforms the regularized non-negative least squares (Reg NNLS) and the sparse recovery-based solver with roughly 80% fewer average reconstruction errors and 50% fewer errors in the standard deviation.

The solver-informed NN applies an existing spectrum reconstruction solver, such as the Reg NNLS solver, to the raw measurements to extract relevant features by training an NN to map the extracted features to the true spectrum. When compared with the plain NN, the average reconstruction error can be further reduced by leveraging the merits of the existing reconstruction solver and the neural network. The combined approach of solver-informed NN can significantly improve the reconstruction performance to achieve higher reconstruction accuracy and stability on synthetic dataset and real datasets collected by the CQD spectrometer. Qualitatively, the average reconstruction error of both Reg NNLS and sparse-recovery-based solver-informed NNs is further reduced by 40% as compared with the plain NN.

The same group also proposed to utilize a denoising autoencoder (originally developed for imaging processing) as one pre-processing technique to the corrupted measurements, and then reconstruct the spectra with the denoised data via a sparse-recovery algorithm [37]. The experiments demonstrate that compared with its non-denoised counterpart, the reconstruction quality can be greatly enhanced by the proposed approach when the raw measurement is noisy under different levels. The results demonstrate that the proposed combination of DNN and reconstruction algorithms holds great promise to deliver a more accurate and stable reconstruction performance for filter-based miniature spectrometers under noisy measurements.

### 3.3 All data-driven spectrum reconstruction

**Figure 7:**
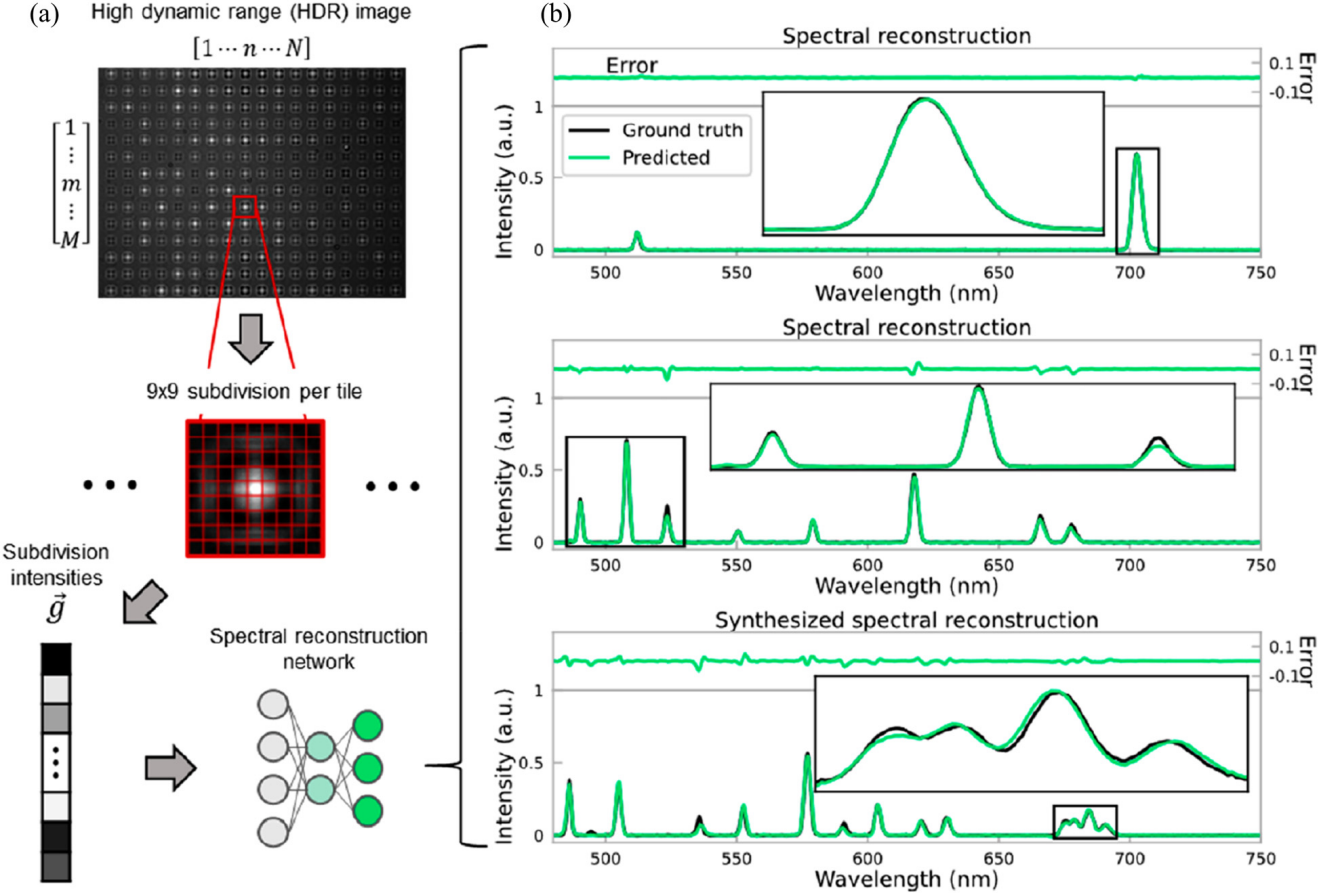
The application of a plasmonic encoder chip enabled on chip spectroscopy. (a) Workflow of spectral reconstructions. Regions of the HDR image corresponding to each tile are used as inputs to the spectral reconstruction neural network. (b) Spectra reconstructed during blind testing. Error is shown above each plot on the same y-scale. The network was trained only on spectra with up to eight peaks, yet it successfully reconstructs a spectrum with 14 peaks. (a) and (b) are reprinted with permission from Ref. [38], copyright 2021 American Chemical Society.

The working principle of a pure data-driven computational spectrometer based on plasmonic encoders is shown in Figure 7 [38]. A trained DNN is used to reconstruct the unknown input spectra from the lens-free diffraction images in a feed-forward manner without the need for *a priori* information on the encoding operation or the input illumination. The training data set consisted of 50,352 spectra, whereas the validation data set consisted of 8824 spectra. When blindly tested on new input spectra of varying complexity captured after the training phase, the DL based spectrometer correctly identified 96.86% of the spectral peaks with a peak localization error of 0.19 nm, a peak height error of 7.60%, and a peak bandwidth error of 0.18 nm. When compared to a linear regression model with L_2_-norm regularization trained on the same data set, the trained neural network achieved a reduction in average MSE from $3.85\times 10^{-4}$ to $7.77\times 10^{-5}$ . These results prove that pure data-driven DNN can be used for highly accurate spectrum reconstruction. However, the authors also discovered that new spectra captured ∼5.8 days after the last training/validation spectrum degraded in performance. To tackle this issue, the authors implemented a transfer learning approach, where the weights of the previously trained neural network were adjusted through further training on a small fraction of the spectra captured at the start of the new measurement period. All performance metrics were significantly improved after the transfer learning step. In addition to these considerable improvements in spectral inference metrics, background spectral noise and erroneous peaks were also suppressed well after the transfer learning step. Therefore, transfer learning can be an effective software-based calibration tool for future data-driven computational on-chip spectrometer. By leveraging such batch computation, the network generates spectral reconstructions in ∼28 μs per spectrum, which is orders-of-magnitude faster than other computational spectroscopy methods. These performance metrics demonstrate significant improvements compared to earlier generations of computational spectrometers despite visible fabrication defects in the plasmonic encoder chip, illustrating the robustness of the DNN-based spectral reconstruction method.

### 3.4 DNN co-design of encoder and decoder

**Figure 8:**
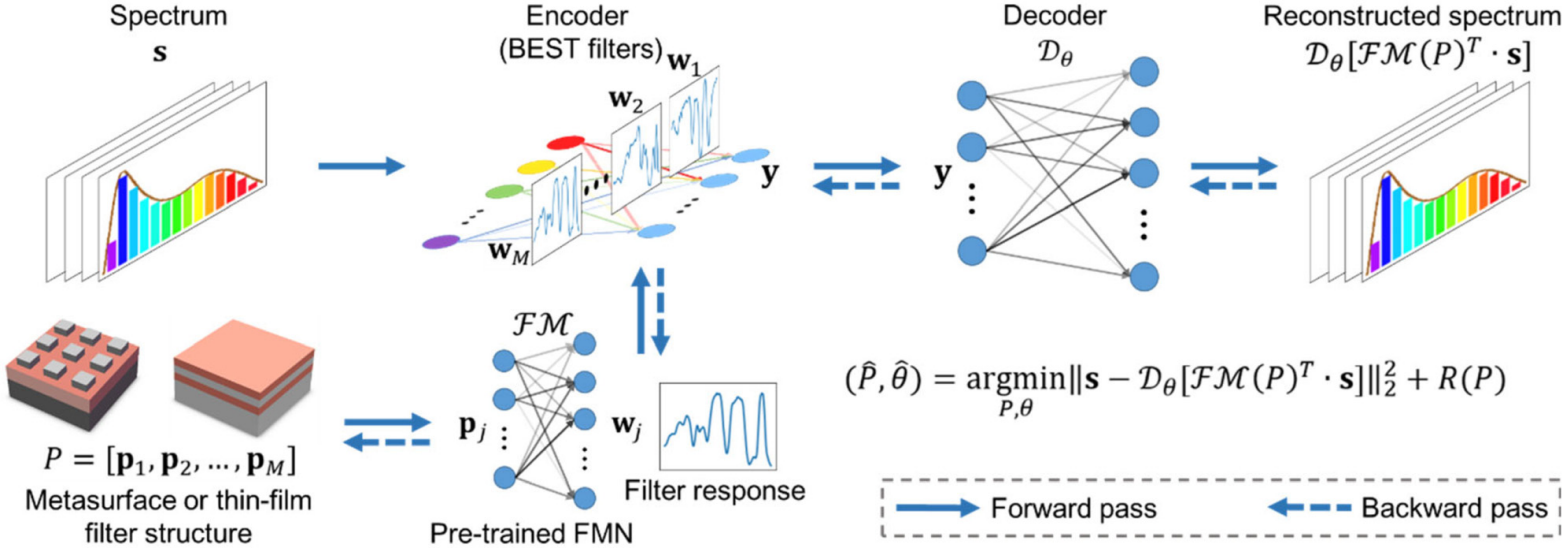
Schematic of parameter-constrained spectral encoder and decoder design framework. The random optical filters act as an encoding neural network whose connection weights and filter responses are constrained by the filters’ structure parameters through a pre-trained forward-modeling network. During each training epoch, a batch of spectrums is fed into the encoder and the decoder gives the corresponding output, such as the reconstructed spectrums. The loss function, such as the mean square error for the reconstructed spectrum, is evaluated, and the errors are propagated back to the structure parameters. Under this framework, the encoding filters and the decoder neural network are jointly designed. Figure is reprinted with permission from Ref. [106], copyright 2021 WILEY-VCH Verlag GmbH & Co. KGaA, Weinheim.

So far, nanophotonic encoding filters in computational spectrometers discussed here are built on a conventional filter design and selection strategy, where sufficient filters are produced to ensure randomness. A filter set is selected via permutation and combination to ensure a relatively low correlation coefficient and allow efficient encoding operation. The encoding potential of optimally designed filters have not been explored. It would be ideal if fewer spectral filters are used to improve spatial resolution for hyperspectral imaging while ensuring the diversity and low correlation of filters for good spectral resolution. The profound inverse-design capability of DL tools should effortlessly empower better intelligent filter design. A novel DNN-based co-design of nanophotonic filters and decoders in computational spectrometers was proposed [106] and is shown in Figure 8. Through the innovative algorithm, metasurface and thin-film types of filters form an encoding network that contains a single fully connected layer. By attaching a decoder behide it, the whole network is named the ‘spectral encoder and decoder’ (SED) it captures and reconstructs the input spectrum. Training the SED grants access to the optimized spectral response functions. By adding regularizations to limit the desired filter parameter range, the design can also be tolerant to fabrication errors. The complete algorithm is referred to as a parameter constraining the spectral encoder and decoder (PCSED) neural network.

This co-design framework allows the structure parameters of filters to be directly trained and leverages back-propagation to obtain the learned structure parameters. Thus it is more convergent than the sequential-inverse design methods for the same task. To compare it with a sequential-design paradigm used for the same task, an SED is built with an L_2_-norm-based smoothness regularization to the encoder [99]. After training the SED, the trained target spectral responses are fed into an inverse DNN to derive the structures of metasurface filters. This comparison shows the co-designed DNN framework can provide more effective spectral responsivity for each random spectral filter than decomposed sequential-design methods. Spectral cameras that use metasurface- and interference-thin-film-based filters present higher reconstruction accuracy with up to 30 times enhancement and protect against fabrications errors more effectively.

**Figure 9:**
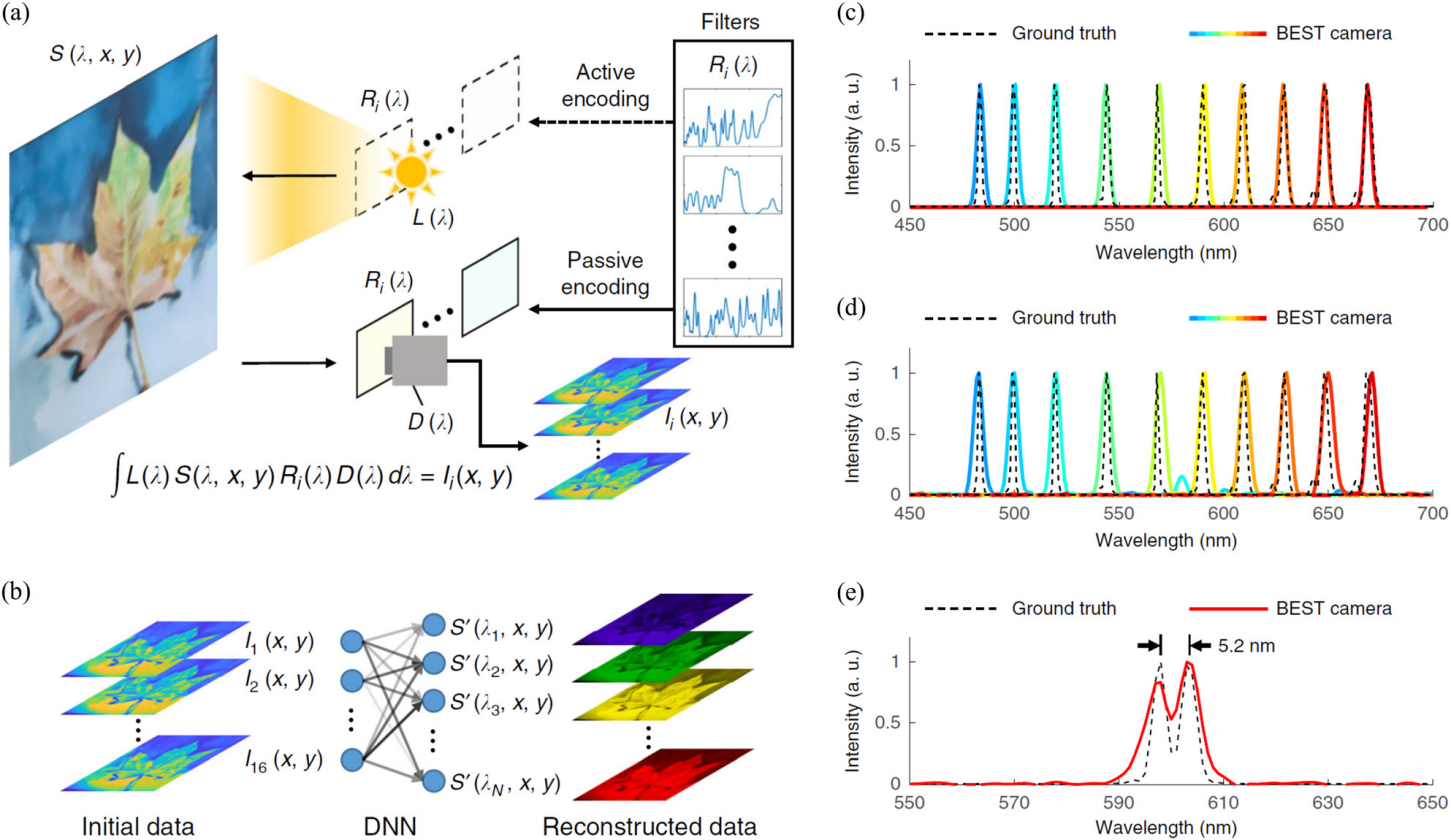
Principle and performance of broadband encoding stochastic camera. (a) Simplified schematic. Depending on where the light spectrum is encoded, the camera can work either in the active (upper) or passive (lower) modes. (b) Principle of DNN-based spectral reconstruction algorithm. The initial data captured by the monochrome camera is fed into the DNN and outputs the reconstructed 3D hyperspectral data cube. (c) and (d) Spectral profiles of laser beams with narrow bandwidth. In (c), the DNN is trained by the “precise” dataset, whereas (d) is for the results from the DNN trained by “general” datasets. (e) Spectral profile of two peaks corresponding to 598.0 and 603.2 nm. The peak-to-peak distance is highlighted in black. In (c–e), the ground truths and the DNN-reconstructed results are represented by dashed (ground truth) and solid (reconstructed) curves, respectively. The graphs are normalized to their peak intensity. (a–e) are reprinted with permission from Ref. [39], copyright 2021 Nature Publishing Group.

Based on this strategy, a simplified schematic of the proposed spectral camera was built using 16 random spectral filters to use in reconstructive hyperspectral imaging tasks. The operation scheme of this novel camera, which has both active and passive modes, is illustrated in Figure 9. The passive mode is the more common mode of operation: the spectrum reflected by the sample is encoded by the filters and the device detects the spectral radiance of the reflected light. In the passive mode, the authors used the camera to acquire a hyperspectral image from a standard color calibration card. The MSE between the measured and ground truth spectra ranged from 5 × 10^−6^ to 0.0032, with an average value of 0.0008 for different colors. For natural objects such as plants, the hyperspectral camera could also reconstruct the spatio-spectral information accurately, with an average reconstruction MSE of 0.0016. In the active mode, the illumination spectrum is filtered first and then encodes the sample; thus, the device detects the spectral reflectance of the sample. Compared with passive mode, active mode excels at measuring spectral reflectance regardless of the ambient illumination. The active hyperspectral camera is set up by placing a random spectral filter array in front of white light-emitting-diodes to modulate the illumination light. By feeding all captured images into the pretrained DNN, its hyperspectral image – a 640 × 480 × 301 3D matrix – is captured. The results cover the entire visible range (400–700 nm) at a step size of 1 nm. The MSE between the measured and ground-truth spectra was quantified at an average MSE of 0.0010. As a comparison, the spectra reconstructed by the CS algorithm have an average MSE of 0.0061, which is worse than the DNN results.

### 3.5 Deep plug-and-play priors for spectral snapshot compressive imaging

**Figure 10:**
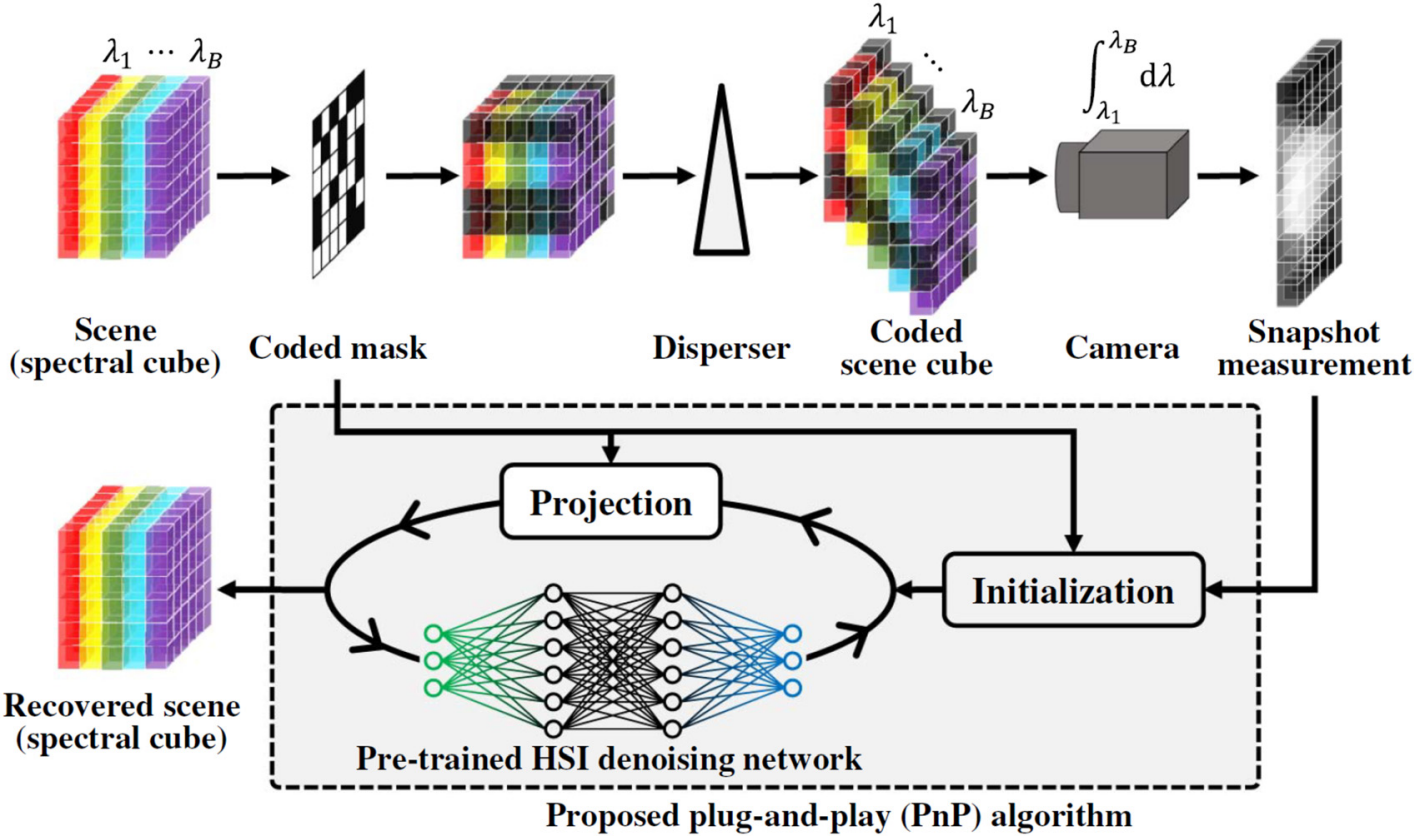
Image formation process of a typical spectral snapshot compressive imaging system, i.e., single disperser coded aperture snapshot spectral imaging and the reconstruction process using the proposed deep PnP prior algorithm. Figure is reprinted with permission from Ref. [119], copyright 2021 Optical Society of America.

As discussed earlier, the reconstruction algorithm plays a critical role in reconstruction spectral imaging as it outputs the desired data cube. The main bottleneck of previously developed iterative optimization-based algorithms is the slow reconstruction speed. The spectral data cube is usually large-scale, requiring hours to reconstruct from a snapshot measurement. To address the low efficiency of reconstructive algorithms, CNN approaches have been used to improve the reconstruction speed [107–111]. These networks can lead to better results than their computational counterparts if sufficient training data and time are given. In general, these networks are usually trained and used for specific problems. More recently, the plug-and-play (PnP) framework [112, 113] has been proposed for inverse problems [114, 115], employing advanced deep denoisers [116–118] in the iterative optimization algorithm to speed up the reconstruction process. In such a framework, no training is required for different tasks, and the same denoising network can be used directly in different systems. This strategy inspired a new PnP method that uses DL-based denoisers as regularization priors for reconstructive spectral imaging, as shown in Figure 10 [119]. This work proposed a CNN-based deep spectral denoising network as the spatio-spectral prior, which is flexible in terms of data size and input noise levels. The proposed denoising network is trained on the CAVE data set, and PyTorh and Adam are used for implementation and optimizer, respectively. Different spectral imaging systems have different settings and parameters for CNN methods, such as λ-net. A different network needs to be trained for each system with large training data and computing resources. However, the PnP method offers flexibility here and can easily be applied to different systems. The actual application of PnP algorithm in three coded aperture snapshot spectral imaging systems, one multispectral endomicroscopy, and a ghost spectral compressive imaging system, shows finer detail and a clean background compared to other algorithms.

### 3.6 Optical diffractive network for spectral encoding

**Figure 11:**
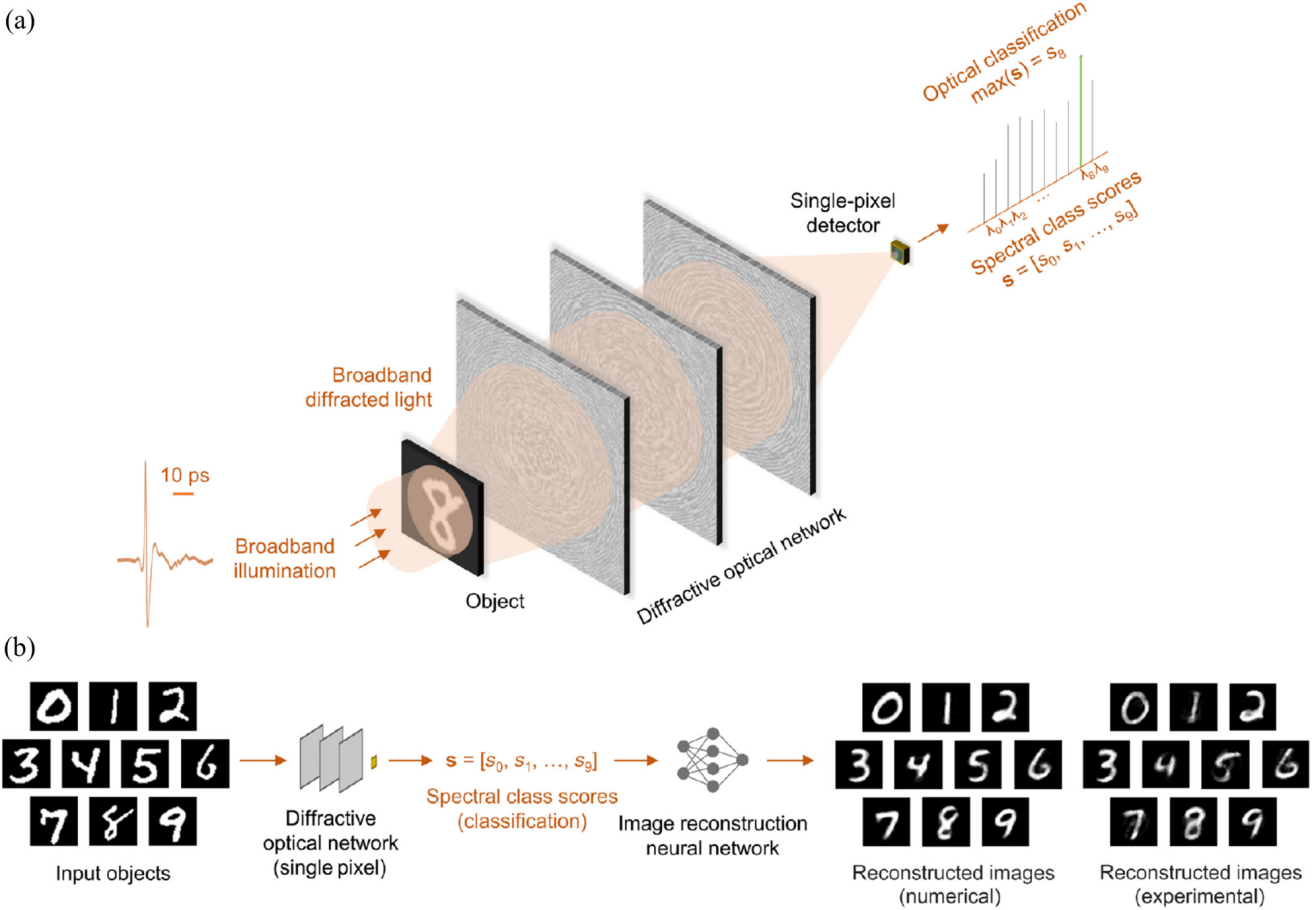
Schematics of spectral encoding of spatial information for object classification and image reconstruction. (a) Optical layout of the single-detector machine vision concept for spectrally encoded classification of objects. As an example, a handwritten digit eight is illuminated with a broadband pulsed light, and the subsequent diffractive optical network transforms the object information into the power spectrum of the diffracted light collected by a single detector. The object class is determined by the maximum of the spectral class scores, s, defined over a set of discrete wavelengths, each representing a data class. (b) Schematic of task-specific image reconstruction using the diffractive network’s spectral class scores as input. A separately trained shallow artificial neural network recovers the images of handwritten digits from the spectral information encoded in s. Each reconstructed image is composed of >780 pixels, whereas the input vector, s, has 10 values. (a) and (b) are reprinted with permission from Ref. [120], copyright 2021 AAAS.

There is growing interest in optical computing to benefit from the low power, low latency, and scalability of passive optical systems. Specially designed light–matter interaction can lead to all-optical processers and perform specific computation tasks through wave propagation. The optical diffractive layers are composed of passive pixels, where the complex-values transmission or reflection coefficients of each pixel are optimized though DL algorithms. It has been demonstrated that DL-based diffractive networks can encode the spatial information of objects into the optical spectrum through learnable diffractive layers that collectively process the information contained at multiple wavelengths [120]. Such systems can perform optical classification of objects and image reconstruction using a single-pixel detector, as shown in Figure 11. A broadband diffractive network composed of layers is trained to transform the spatial information of the objects into the spectral domain through a preselected set of class-specific wavelengths measured by a single-pixel spectroscopic detector at the output plane. The resulting spectral class scores are denoted by the vector s = [s0, s1, …, s9]. After its training and design phase for a given input/test image, it learns to channel relatively more power to the spectral component assigned to the correct class, e.g., digit “8” in Figure 11a; therefore, max(s) reveals the correct data class. As demonstrated in Figure 11b, the same class score vector, s, can also be used as an input to an NN with two hidden layers to reconstruct an image of the input object, decoding the spectral encoding performed by the broadband diffractive network. Even if the input objects are incorrectly classified by the trained diffractive network, by feeding back the incorrectly reconstructed images into the same network as new inputs, their optical classification is corrected with significantly improved inference accuracy. This framework demonstrates broadband diffractive optical networks that operate with pulsed illumination at terahertz wavelengths to achieve >96% blind testing accuracy for optical classification of handwritten digits based on the spectral power at 10 distinct wavelengths, each assigned to one digit. The authors also experimentally verified the results by using a terahertz time domain spectroscopy system and 3D-printed diffractive models. These results emphasized the vital role of collaboration between a trainable optical front end and an all-electronic NN-based back end.

## 4 Discussion

### 4.1 Spectrum reconstruction quality

Previous iterative reconstruction methods rely on precise measurements of the encoding operation and the spectral response of the underlying photodetectors, which are both used as *a priori* information. This presents additional challenges, as the reconstruction performance relies on how precisely one can characterize the underlying hardware when system noises are present. In addition, the number of filters also plays a critical role in successful spectral reconstruction. In earlier FB-resonator-based works [20, 61], M = 23 or 100 filter response functions are used. The total number of filters sets a limit on the spectra that can be successfully reconstructed, and this limit is related to the sparsity of the measured spectrum. Laser spectrum that is sparse in wavelength is well-suited for compressive sensing, while a larger number of filters are needed to reconstruct spectra that are less sparse. The adoption of filter response functions with broadband and random resonance features have indicated that an array of 42 PC filters can reconstruct more accurately than an array of 9 PC filters [23]. The investigation on the effect of the number of metasurface units M in a single spectrometer also reveals that increasing M can decrease reconstruction error, as it reduces the randomness of the solution to the matrix equation. Based on the noise level, M can be dynamically adjusted based on the reconfigurable metasurface supercell to achieve an optimal trade-off between density and reconstruction quality. In other words, a small M and large spectral pixel density could be used at low noise levels, whereas a large M, with degraded spectral pixel density could be used at high noise levels [27].

In addition, some earlier iterative reconstruction algorithms employ the smoothness constraint in their optimization process. The selection of a singular weighting parameter on this constraint usually introduces a trade-off in performance between narrow-band and broad-band spectral reconstructions. However, this is not a challenge if one uses DL-based spectroscopy approach. Therefore, the reconstructive spectrum can obtain ultrahigh spectral resolution, as seen in summarized Table 1. The resolution of spectral reconstruction networks is primarily limited by the spectral resolution of the instrument used for ground truth measurements of the training data. The best resolution a computational spectrometer can achieve is below 1 nm. The reconstructed spectral resolution is comparable for both CS computational algorithms and DL based approaches. In direct comparisons, all methods of TR, CS, and DL perform well in the oversampling regime, while in the undersampling case, DL yields a very good performance and even clearly outperforms CS for dense spectra [103]. One major weakness of utilizing such spectrometers for hyperspectral imaging is that the spatial imaging resolution is limited by the spectrometer pixel size. However, this issue can be continuously improved with better filter design with fewer numbers of filters and by scaling down the single filter size to match the single detector pixel size. A recent study of snapshot ultraspectral imaging has proved that 6336 microspectrometers containing a total of 154,800 metasurface units can be integrated with CMOS image sensors [27]. The adoption of diffuser can spatially multiplex the information and thus improve the spatial resolution as well [83].

**Table 1:** The performance comparison of some recent computational spectrometers.

Filter structure	Reconstruction method	Detector type	Spectral range (μm)	Spectral resolution (nm)	Spatial resolution (μm)	Ref.
Plasmonic structure	TR	CMOS	0.350–1.05	10	–	[32]
Random photonic chip	CS	–	1.5–1.525	0.75	–	[13]
Colloidal quantum dot	CS	CCD	0.39–0.69	3.2	–	[19]
Elaton array	CS	CCD	0.4–0.75	8	–	[20]
Fabry–Perot mirror	CS	–	0.5–0.9	1	–	[62]
Nanowire mat	TR and CS	CMOS	0.61–0.67	1	2.4	[26]
Plasmonic metasurface	RLS	IR microscope	1.5–1.9	–	–	[21]
Photonic crystal slab	CS	CMOS	0.55–0.75	1	200	[22]
Photonic crystal slab	CS	CMOS	0.4–0.7	1	–	[23]
Single nanowire	Adaptive TR	Paired nanowire	0.5–0.63	15	50	[65]
Voronoi photonic crystal	Demosaicking	–	0.42–0.72	10	232.5	[68]
Colloidal quantum dot	NN	CCD	0.4–0.75	5	–	[36]
Metasurface	CS	CMOS	0.45–0.75	0.8	80	[27]
Pearl	CS	–	0.45–0.7	7.4	–	[64]
Plasmonic nanohole array	NN	CMOS	0.48–0.750	0.229	100	[38]
Colloidal quantum dot	NN	CCD	0.4–0.75	5	–	[37]
Multilayer film	NN	CCD	0.4–0.7	5.2	–	[39]
Black phosphorus	TR	Black phosphorus	2–9	420	–	[70]

### 4.2 Spectrum reconstruction speed

The key advantage of the data-driven approach is its speed compared to common spectral reconstruction algorithms based on least-squares minimization. Previous study observes that although solver-informed NN requires additional computational cost to run existing spectrum reconstruction solvers for each spectrum to generate training input, it is still advantageous in terms of overall speed, as the time-consuming fine selection process for regularization parameters is eliminated [36]. The major burden for all data-driven reconstruction processes is the training data collection. Although the DNN training process requires a large number of measured data sets, this is a one-time effort. After the training is complete, the trained model can blindly recover unknown spectra from raw sensing signals in microseconds – several orders of magnitude shorter than the time required to solve iterative minimization problems employed in earlier spectral reconstruction methods [38]. This efficiency leap is consequential because DNN operates matrix multiplexing and parallel computing while the iterative algorithm solves optimization problem sequentially for each pixel. This is especially true for computational algorithms, as the reconstruction time of a spectral image increases with increasing numbers of pixels. However, the reconstruction speed of the DL is almost pixel-independent, and is more advantageous for images with higher resolution.

For applications such as hyperspectral imaging, which may demand a spectral recovery across a large sequence of images, each with a large number of individual pixels, DL could be the only viable choice. In general, the speed of DNN is almost irrelative to the spatial resolution, while the consumption time of the CS algorithm and the number of pixels follow a roughly linear relationship. Running iterative CS algorithms is inefficient on GPU because frequent data exchange is needed for iteration. The comparison study between DL and CS algorithm in Ref [39] shows that to reconstruct a hyperspectral image (480 × 640 pixels, 1 nm spectral step size, 400–700 nm spectral scale) on an Intel Core i9-10900x CPU, Nvidia GeForce 2080Ti GPU platform, the DNN could generate such an image in 0.48 s. Reconstructing the same image using the basis pursuit denoising algorithm requires 3307.3 s on the same computer platform – nearly 7000 times slower than that of the proposed DNN. Although further optimization is possible, the current speed of DNN is sufficient to perform real-time data reconstruction, as the proposed recording frame rate is below 2 fps. DL-based hyperspectral reconstruction is suitable for real-time image processing. DL reconstruction speed may still be increased via performance optimization of the code, the implementation of multi-GPU platforms, and training networks that reconstruct speckles in parallel.

### 4.3 Spectrum reconstruction denoising

In CS algorithms, however, denoising is more experience-dependent, as the selection of the non-negative parameter influences the reconstruction accuracy under noisy conditions. In general, a variable parameter is manually set during the iteration to cancel the noise-induced bias. This operation works to some extent, but does not provide convincing results when the noise level changes. As examined, the DNN enhances noise tolerance by 8.14 times under different noise levels [39], even compared to the CS algorithm with optimal parameter settings. In a real-life scenario for spectrum reconstruction, there is shot noise, electronic camera noise or other non-specific backgrounds present. An imaging system may also experience some drift caused by environmental effects such as vibrations and thermal variations [103]. On the other hand, the use of a denoising autodecoder shows the flexibility of DNN to deal with spectrum data measured under noisy environments [37, 117, 118]. More importantly, well-trained DNN has better denoising abilities, which can be readily obtained by adding more samples with noise at different levels during training. Moreover, adding noise has a regularization effect and further improves the robustness of the model. To assess the robustness of the different approaches against typical perturbations, performance robustness of DL, CS, and TR is compared in speckle reconstruction. As CS and TR are based on analytic methods that are intrinsically inflexible to variation, DL has the advantage in adapting to perturbed data and noise through the choice of proper training data. Noise-adapted DL shows a distinct performance boost at reconstruction from non-perfect speckle images. The training set might not only contain data accounting for shifts of the CCD, but also for shifts of fiber or variations in the incident angle of light. All these effects affect the speckle patterns in a deterministic way and could be corrected with properly trained DNN, while this is impossible with an analytical method [103].

### 4.4 Potential applications for computational sensing

**Figure 12:**
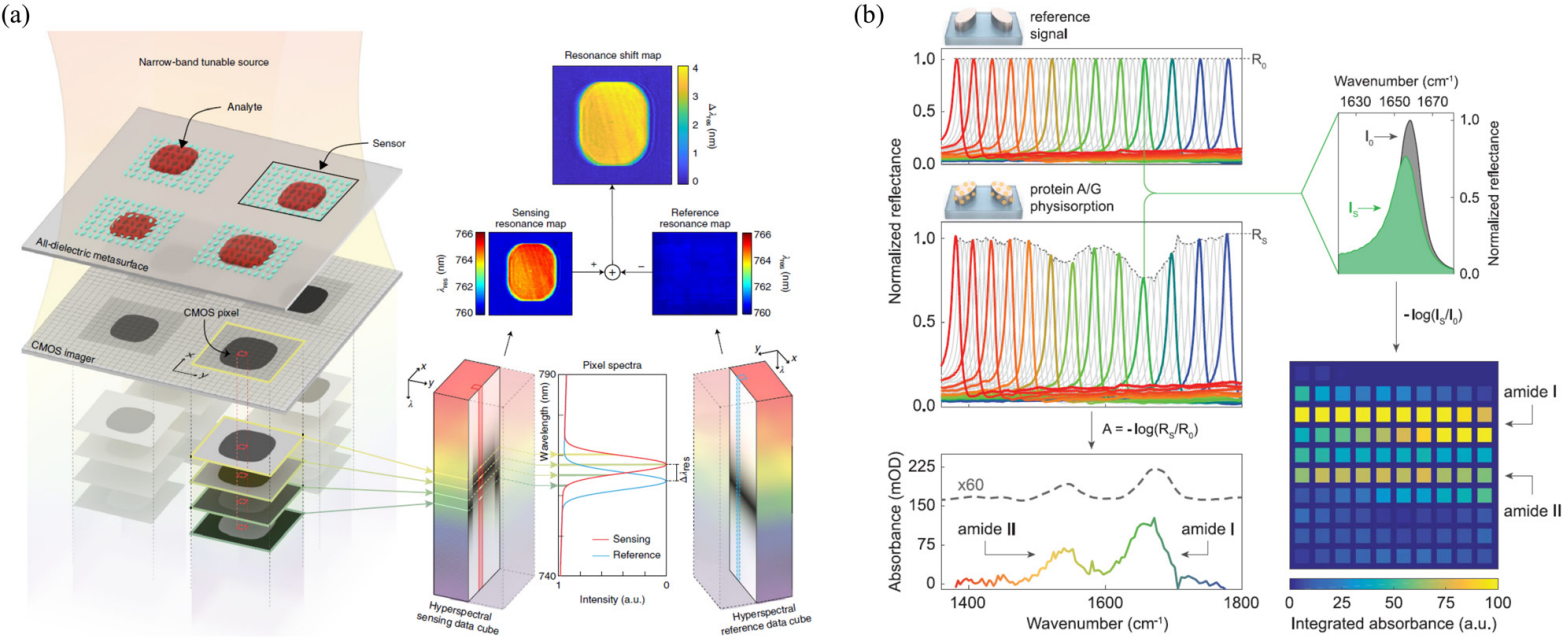
Hyperspectral imaging-based biomolecule detection using all-dielectric metasurfaces and molecular fingerprint retrieval based on spatial absorption mapping. (a) Sketch of the hyperspectral imaging principle showing a representative dielectric metasurface sensor array illuminated with a narrow-band tunable laser source. At each wavelength illumination, images are recorded by a CMOS camera to create a hyperspectral data cube where each CMOS pixel captures high-resolution spectral information. The hyperspectral data cube is processed to extract [57] spatial resonance maps of each individual sensor. For biomolecule detection, the sensing and reference resonance maps are combined to create the resonance shift map, which conveys spectral shift information across the whole sensor. (b) Normalized metapixel reflectance spectra before and after physisorption of a protein A/G monolayer. R0 indicates the envelope of peak reflectance amplitudes and RS is reflectance envelope RS. Protein absorption fingerprint calculated from the reflectance envelopes R0 and RS compared to an independent IRRAS measurement. Broadband spectrometerless operation of the metasurface can be emulated by integrating the reflectance signal of all pixels. Spectral integration translates the absorption signature into a 2D spatial absorption map that represents the molecular barcode of the protein. (a) is reprinted with permission from Ref. [58], copyright 2019 Nature Publishing Group; (b) is reprinted with permission from Ref. [57], copyright 2018 AAAS.

Since the computational spectrometer can capture spectral information accurately, it can potentially be used as a novel computational sensor [121–128]. Figure 12a introduces a metasurface-based multi-resonance sensor design and a new concept: a hyperspectral decoder that associates spectral information from each CMOS pixel to its spatial index [58]. By employing a pixel-based thresholding method, the detection limit can be improved by three orders of magnitude when compared to ensemble averaging (∼3 versus 1500 molecules per μm^2^). This encoder-decoder scheme eliminates the need for bulky and expensive instrumentation and can be used to realize field-deployable sensors. Similarly, a mid-IR nanophotonic sensor based on all-dielectric high-Q metasurface elements demonstrates its capability for enhancing, detecting, and differentiating the absorption fingerprints of various molecules [57]. The high-Q resonance design is spectrally clean without additional resonance background, which allows for highly spectral-selective enhancement of spectroscopically rich molecular fingerprint information. As the resonance positions of individual metapixels are linearly varied over a target mid-IR fingerprint range, so each resonance position is assigned to a specific pixel of the metasurface, establishing one-to-one mapping between spectral and spatial information. By comparing the imaging-based readout of this spatially encoded vibrational information before and after the coating of target analyte molecules, chemically specific molecular barcodes are proved to be suitable for chemical identification and compositional analysis, as shown in Figure 12b. Although the abovementioned cases are not meant for spectrum reconstruction, they share similar hardware set-up and potential decoding algorithms. In conventional biological or chemical spectral sensing systems, the small resonance shifts caused by analytes are characterized by conventional spectrometers. Computational spectrometer systems have the potential to be used for computational sensing as well, since the system can decode spectral changes with high resolution and thus probe tiny spectral signal shifts caused by analytes on nanophotonic filters. In this case, high Q- and narrow-band nanophotonic filters may be still preferred for sensing as opposed to spectrum reconstruction applications where broadband and random resonant structures are desired.

## 5 Summary and outlook

Although conventional spectrometers can be further miniaturized by scaling down the dispersive optical components through advanced fabrication, computational spectrometer may be a novel and revolutionary technology shift. It has rapidly developed proof-of-concept applications over the past decade. It shows new possibilities of ultra-compact, low-cost spectrometers, and spectral imagers with potential to be integrated with smartphones and lab-on-a-chip systems. Computational techniques possess great advantages compared with conventional optical spectroscopy technology, but are still in their infancy, with significant space for improvement and application expansion. The implementation of various random, broadband nanophotonic filters and DL algorithms have expediated development. DL-based computational spectrometers show prominent advantages compared to CS methods in terms of operation speed, denoising capability, and lack of required precalibration. However, emerging technology like DL-based spectroscopy also has inherent drawbacks and challenges. Copious well-characterized and well-labeled training data needs to be collected, and this could be a great burden for experimental data generation. The data can be easily biased or noised due to system instability over a period of time, and this problem may require better network architecture and training methods.

Nonetheless, efforts will continue as the co-design of hardware and computational algorithm will lead to wider use of spectral information. Maturation of DL algorithms could ease the burden for denoising and speed up the reconstructive process. Moreover, with flexible fabrication and packaging of filters and detectors, the miniature computational spectrometer could be made suitable for wearable devices, as proved in related flexible electronic technologies. Furthermore, bandgap engineering of semiconductor nanowires and two-dimensional materials could offer a route toward printable, conformable nanoscale spectrometers that combine filter and detector functions in a single device. The simultaneous capture of spectral and spatial information in large data cubes for reconstructive spectral imaging could potentially revolutionize existing hyperspectral imaging applications, and most importantly reduce the cost of spectral image cameras. Beyond smartphones and lab-on-a-chip sensing and imaging applications, such high-performance computational spectrometers may also find applications in a vast range of research fields and industrial technologies, including satellites and drones, the food industry, chemical and biological imaging, etc.
